# Microplastics in the Surface Water and Gastrointestinal Tract of *Salmo trutta* from the Mahodand Lake, Kalam Swat in Pakistan

**DOI:** 10.3390/toxics11010003

**Published:** 2022-12-20

**Authors:** Muhammad Bilal, Habib Ul Hassan, Mohammad Abdul Momin Siddique, Wali Khan, Karim Gabol, Imran Ullah, Saira Sultana, Umaiya Abdali, Shahid Mahboob, Muhammad Shahab Khan, Usman Atique, Muhammad Khubaib, Takaomi Arai

**Affiliations:** 1 Department of Zoology, Government College University Lahore, Lahore 54000, Pakistan; 2 Department of Zoology, University of Karachi, Karachi 75270, Pakistan; 3 Fisheries Development Board, Ministry of National Food Security and Research, Government of Pakistan, Islamabad 44000, Pakistan; 4 Department of Oceanography, Noakhali Science and Technology University, Noakhali 3814, Bangladesh; 5 Department of Zoology, University of Malakand, Chakdara 18800, Pakistan; 6 Dr. A. Q. Khan Institute of Biotechnology and Genetic Engineering, University of Karachi, Karachi 75270, Pakistan; 7 Department of Zoology, College of Science, King Saud University, P.O. Box 2455, Riyadh 11451, Saudi Arabia; 8 Department of Chemistry, University of Malakand, Chakdara 18800, Pakistan; 9 Department of Bioscience and Biotechnology, College of Biological Systems, Chungnam National University, Daejeon 34134, Republic of Korea; 10 Department of Zoology, Abdul Wali Khan University Mardan, Mardan 23200, Pakistan; 11 Environmental and Life Sciences Programme, Faculty of Science, Universiti Brunei Darussalam, Gadong BE 1410, Brunei

**Keywords:** MPs pollution, freshwater lake, high altitude, ingestion by fish

## Abstract

Microplastic pollution is becoming an increasingly severe environmental problem. As compared to the marine ecosystem, freshwater ecosystems at high-altitude, remote regions are less studied and lag far behind. Thus, the present study aims to highlight this issue and fill the gap in this regard. The presence of microplastics (MPs) in the surface water and the gastrointestinal tracts (GITs) of brown trout (*Salmo trutta*) from Lake Mahodand, Kalam Swat, Pakistan, at a high altitude of 2865 m above sea level was investigated. For microplastic extraction, samples were digested with H_2_O_2_, NaCl solution was added for density separation, and then samples were filtered with a cellulose nitrate filter (pore size 0.45 µ). After this, visual observation and polymer detection with Fourier transform infrared spectroscopy, microplastics were characterized by their shapes, sizes, colors, and polymer types. In the surface water sample, MP particles were found in the range of 0–5 MPs/L, where the mean concentration of MPs was 2.3 ± 1.52 MPs/L and 1.7 ± 1.05 MPs/gastrointestinal tract (GIT) isolated from the GIT of brown trout. Particles of relatively larger size (500–300 µm) were more abundant than other ranges of particles (300–150 and 150–50 µm) in the surface water and fish samples. The fiber was the most abundant shape of MP particles, followed by sheets and fragments in surface water and fish samples (fibers > sheets > fragments). Four types of polymer viz. low-density polyethylene (LDPE) (44.4%), polypropylene homopolymer (PPH) (19.4%), polyvinyl chloride (PVC) (30.5%), and high-density polyethylene (HDPE) (5.5%) were detected by FTIR spectroscopy. The findings of the present study showed that MPs reached into higher altitudes in remote areas due to tourism activities.

## 1. Introduction

We live in a plastic age in which we use more than 348 million tons of plastic per year [1]. The word plastic refers to materials made up of a broad range of polymers, including polyethylene, polypropylene, polystyrene, polyamide, polyethylene terephthalate, polyacrylonitrile, polyvinyl chloride, and styrene-butadiene rubber [2,3]. Plastic debris accumulates in the environment due to the limited recovery of discarded materials and their durability [4]. Over a third of plastic production is used for disposable packaging items, most of which are discarded within a year or so after manufacture [5,6]. Therefore, plastic has accumulated in natural habitats from the poles to the equator [5]. These larger plastic items disintegrate in the environment through physical fragmentation, photo degradation, chemical weathering, or microbial-mediated biodegradation, leading to microplastic production [7,8]. Fragments of plastic resulting from fragmentations of size (<5 mm) are called MPs [9,10].

MPs have been classified into different types based on source and origin, sizes, and shapes. According to their origin and sources, these MPs are either primary MPs or secondary MPs. Primary MPs come into the environment in particles such as pellets in personal care products, facial scrubs, and other cosmetics materials [11]. The secondary MPs come from the degradation of larger plastic fragments present in the environment [5,12]. Based on their size ranges, particles are termed macroplastics (>5 mm), MPs (1–5 mm), mesoplastics (0.1 µm–1 mm), or nanoplastics (<0.1 µm). Based on geometry, these particles may be fibers, sheets, fragments, foams, and beads [13,14]. Microplastics come from various sources such as household activities, washing clothes, disposable trash, industrial discharge, and wastewater treatment plants, as MPs are not trapped by filters and quickly make their way to the aquatic environment [15,16]. When they contact with living systems, these MPs cause toxic effects and act as a vector for further pollutants such as heavy metals which lead to various forms of toxicity [17,18,19,20,21,22]. Ingestion of MPs has been reported to affect growth rate, block enzyme production, and cause mechanical injury and oxidative stress [23] in aquatic organisms. Invertebrates, fish, birds, and mammals have been studied and reported to have considerable MPs [8]. The gastrointestinal tract (GIT) is the main target of MPs in aquatic animals [24,25,26]. The presence of MPs in the GIT, gills, and stomachs of fishes has been reported in many studies across the globe and confirmed the evidence of plastic ingestion by fishes as well as other aquatic organisms [27,28,29,30]. An assessment of a large number of fish species (427) from different regions, ecosystems, and guilds was carried out in which the authors collected numerous amount of MPs [31]. Microplastics were found in 72% of fish having recovered from 66% of the gastrointestinal tracts (GITs) and 28% of stomach contents [32]. In addition to other fish species, *Salmo trutta* have been reported to ingest MPs in 68% of analyzed individuals [33]. The effects of MPs on *Salmo trutta* embryos and larvae lead to genotoxic responses, and larvae exhibit an increased frequency of genotoxic endpoints after exposure to MPs [34]. The slight effect on the resting behavior of fry was seen in *Salmo trutta* exposed to MPs [35]. Generally, ingestion caused many physical and physiological problems in these aquatic organisms. Among these possible problems inside the gastrointestinal tract is the covering of villi in the intestines, the blocking of various digestive enzyme secretions, and other hormonal issues [36]. Furthermore, humans are not safe either, and these MPs can cause a hazardous effect on human health via direct inhalation, ingestion, or through the food chain [37].

Freshwater and sediments have been reported globally to contain MPs, but fewer reports are available on MPs in freshwater fishes and other organisms [38,39,40,41,42,43,44]. Many of the latest studies have given attention to microplastic (MP) pollution in the freshwater ecosystem [45,46,47,48,49]. For the chemical composition of the polymer type of these MPs, many studies have been carried out with Fourier transform infrared spectroscopy (FTIR). This is the most popular approach for chemical identification nowadays and simply works on a species-specific frequency absorbance of IR radiation [50]. However, very limited studies have detected polymer types of different MP particles and been published in Pakistan. At higher altitudes, remote regions are also vulnerable to MPs pollution due to anthropogenic activities such as tourism and mining, etc., which ultimately lead to lowland downstream pollution and also, finally, a major proportion of plastic load to the sea. Mahodand Lake was formed by melting glaciers and springs from the Hindu Kush mountains. The lake is a tourist hub and is mostly occupied by tourists; according to data released by the government, 2.7 million tourists visited this area during the Eid festival (Tourism Department Khyber Pakhtunkhwa, 2021). Therefore, the main objective of this study was to investigate the presence of MPs in the gut of *Salmo trutta* and the surface water of Lake Mahodand, Kalam Swat, Pakistan.

## 2. Material and Methods

### 2.1. Study Area

**Figure 1:**
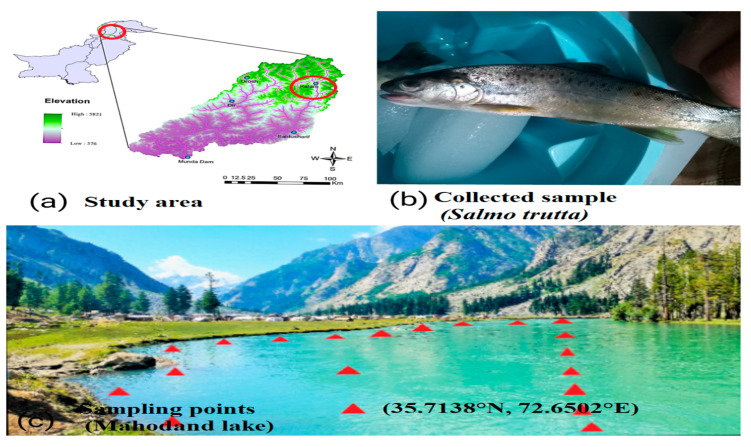
The study area map “(**a**)” sampled fish species “(**b**)” and sampling locations of collected fish and surface water samples “(**c**)”.

Mahodand Lake (35.7138° N, 72.6502° E) is an international tourist spot in Khyber, Pakhtunkhwa Province, Pakistan. It is 35 km away from the Kalam with an altitude of 2865 m above sea level, a length of 2 km, a width of 1.2 km, and it is surrounded by grassland, mountains, and dense forest. *Salmo trutta* is the dominant fish species in addition to *Channa gachua* (the snakehead fish) and *Schizothorax plagiostomus* (Swati fish). Brown trout were selected for the current study due to their high economic value and high demand in the local market (Figure 1).

### 2.2. Sampling and Laboratory Treatment

During the summer season (June and July) of 2019, 20 surface water samples were collected from shores and different spots of the same lake by using a local tourist boat with a 1-L glass jar. The lid was removed, and then the glass jar was dipped into the water taken just one inch below from the surface. When the jar was filled, it was recapped and stored, and then ten individuals of the brown trout with almost identical body lengths (mean 20 ± 4.19 cm) were caught with the help of local anglers. All samples were stored in an icebox and transported to the laboratory for further analysis. Digestion of organic material in the water samples was carried out to eradicate any organic material (plant litter or insect body parts) present in samples by using 20 mL of hydrogen peroxide (35% conc.) and 20 mL of Fenton’s reagent for each 200 mL of the sample [51,52,53]. After adding these chemicals, samples were kept on a hot plate at 75 °C to accelerate digestion. After digestion, a concentrated NaCl solution with a density of 1.18 g/cm^3^ was used to separate MPs [51,54]. For density separation, a 600-mL solution of NaCl was poured into the digested water sample and kept for 8 h for the settlement. After that, the filtration was done, and the supernatant layer from the sample was filtered through a series of sieves of 500, 300, 150, and 50 µm to get fractions of each sample. The contents of each sieve were collected on filter paper (MCE 0.45 µm pore size and 47 mm diameter) by using a vacuum filtration assembly [55].

Ten fish samples were dissected to collect the GIT. The GIT was separated, weighed, and stored in 250-mL glass bottles for digestion. A total of 10% of KOH solution was added to each sample to digest the fish GIT tissue [21]. The KOH solution and fish GIT ratio were maintained at 5:1 and incubated at 55 °C for 36 h. After digestion, no visible organic materials were found, and then sodium chloride (3:1 *v*/*v*) was added to the sample and stirred for 20 min. The sample was set for density separation for 2 h, [56]. The upper layer supernatant was separated and filtered through 500-, 300-, 150-, and 50-µm sieves. By using vacuum filtration assembly, the solid contents of these four types of sieves were transferred to filter paper (MCE 0.45 µm pore size and 47 mm diameter). Finally, the filter papers were kept in a petri dish for drying at room temperature and covered with aluminum foil. After 24 h, the sample was ready for detecting MPs. All precautionary steps were made to control atmospheric contaminations in samples. The samples were processed in a dedicated place in the laboratory, and all glassware and chemicals were covered with aluminum foil when not in use. To prevent contamination from the atmosphere, reagents and distilled water were also filtered and wrapped with aluminum foil. To measure the suspended load of MPs from the laboratory environment, a few filter papers were placed in a few different locations throughout the lab for 72 h. These filter papers were then examined under a stereomicroscope. For the duration of the analysis, six of these filter papers were evaluated and preserved as a control.

### 2.3. Detection of MPs

Dried filter papers containing solid contents were examined, and the MPs were visually observed at 16 × 4, and 16 × 10 magnification by using a light binocular microscope (Labomed, model: CXL-110446002, 9135002, NY, USA and images of plastic items were taken with a Zeiss stereomicroscope stemi 508 microscope at 2.5× magnifications and 1600X USB 8 LEDs electronic digital microscope camera. The lower limit of MP detection was 50 microns; any particle less than 50 microns was not considered in the current study. As per the physical characteristics, the morphotypes of obtained MPs were classified into fragments, fibers, foams, beads, and films [55]. The colors and physical shapes of the MPs were used for category identification. For the identification of the plastic nature of the particles, hot needle tests were done for all samples and confirmed their plastic nature before counting them. For polymer detection, FTIR spectroscopy (IRTracer-100, Shimadzu, Columbia, MD, USA) was used. The MP particles from the water and fish samples were placed into the FTIR, which was appropriately programmed to collect the sample spectrum. After receiving the selected sample spectrum, polymer identification was made by using the polymer spectral library of Omnic Spectra (Thermo Fisher Scientific Inc., Waltham, MA, USA) software.

### 2.4. Data Analysis

All data were analyzed by using Microsoft Excel 2010. The MPs were identified and categorized according to their shape and texture, according to [21]. The length size of the MPs was determined by using ImageJ software.

## 3. Results and Discussion

**Figure 2:**
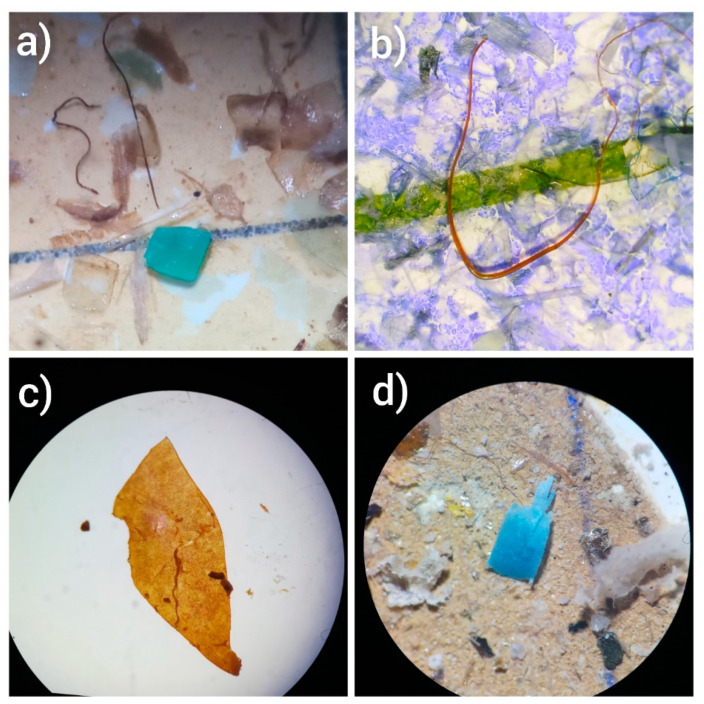
Microscopic images of some of the extracted MPs representing different particle shape, (**a**,**d**) fragments, (**b**) fiber, and (**c**) sheet.

In the present study, MPs larger than 500 µm were not detected from the surface water and fish GIT samples. A total of 46 particles were isolated from 20 surface water samples of the Mahodand Lake. Microplastic particles were found in the range of 0 to 5 MPs/L in the surface water samples, where the mean concentration of MPs was 2.3 ± 1.52 MPs/L. In the GIT of fish, a total of 17 particles of MPs were isolated from the GIT of 10 trout. Detected MP particles were 0 to 3 MPs/GIT, and the mean concentration was 1.7 ± 1.05 MPs/GIT (Figure 2 and Table 1).

The current study found MP concentrations in the range of published reports across the globe. A previous study has reported 3.12 to 11.25 MPs/L in the surface water from one of the northern areas of China’s Wuliangsuhai Lake [57]. Another study reported an average of 6.33 ± 2.67 MPs/L in Gehu Lake in southern Jiangsu, China [58]. The mean concentrations of MP were reported as 3.67 to 10.7 MPs/L in Wei River, China [59]. Moreover, 4 to 26 MPs/L from the Lakes in Siberia, Russia [60] were reported. By contrast, Lake Ontario in Canada was assessed for MP pollution and 0.8 MPs/L was found in the surface water, which was very low compared to our present report [61]. The sample processing protocol of the study to which the current study has been compared was almost the same with some minor modifications, namely that they used the same chemicals hydrogen peroxide for organic digestion, NaCl for density separation, and filtration of the supernatant containing MP particles with filter papers or sieves and finally observation with a microscope, except for [60], in which no organic digestion of water samples was done. The reason for this might be due to the presence of crystal-clear water with no organic matter that requires digestion. Generally, the concentration of MPs in the surface water depends on anthropogenic activities in the vicinity of the water bodies and catchment area, the number of industries, and fishing and tourism activities, etc. Lake Mahodand is an international tourist spot, and the primary source of microplastic load comes from tourism activities in the form of food wrappers, disposables, and fishing activities. Agriculture is also prominent in the vicinity, and due to the high rush of the tourist market, people are active and are responsible for the plastic load.

Furthermore, MPs in fish GIT of the current study (0 to 3 MPs/GIT with a mean concentration of 1.7 ± 1.05 MPs/GIT) were very close to previous reports. One study reported a mean concentration of 1.7 MPs/GIT in the *Piaractus brachypomus* from Vembanad Lake, the largest brackish water lake on the southwest coast of India. In their study, 69 particles were separated from 32 guts of 123 fish (26%) [62]. The present study found 17 particles in the guts of 9 out of 10 fish (90%). Ref. [63] reported 1.24 MPs/GIT from the Como Lake and 5.59 MPs/GIT from Lake Garda, Northern Italy, in *Perca fluviatilis*. From the Irish riverine system, 1.88 ± 1.53 MPs/GIT have been recovered in GIT of *Salmo trutta* which is in a comparable range with the current study [64]. Another study has reported the MPs with a range of 1 to 6 MPs/GIT in eight different fish species from a municipal water supply lake (Eleyele) in Nigeria [65]. Several findings have reported MPs in the fish guts from different aquatic ecosystems, with 5.50 MPs/GIT in the lake fishes, 5.46 MPs/GIT in the estuarine fishes, 2.91 MPs/GIT in the river fishes, and 2.85 MPs/GIT in the marine fishes [66]. A deficient concentration of MP was reported in the fish GIT (0.41 MPs/GIT) in *Cyprinus carpio* and *Alburnus mossulensis*, from Sürgü Dam Reservoir, Turkey, and a higher concentration of MP was found in the fish GIT from Lake Ontario, Canada in seven different species of fish (59 MPs/GIT) and Lake Van Turkey (34 MPs/GIT in *Alburnus tarichi*) [67,68,69]. The methodology of fish sample processing was the same as all the compared studies, in which they used KOH solution for GIT digestion and NaCl for density separation. The possible reason for this fluctuation in concentration might depend on various factors, including the pollution profile of water and anthropogenic activities in the surrounding or lake water.

**Figure 3:**
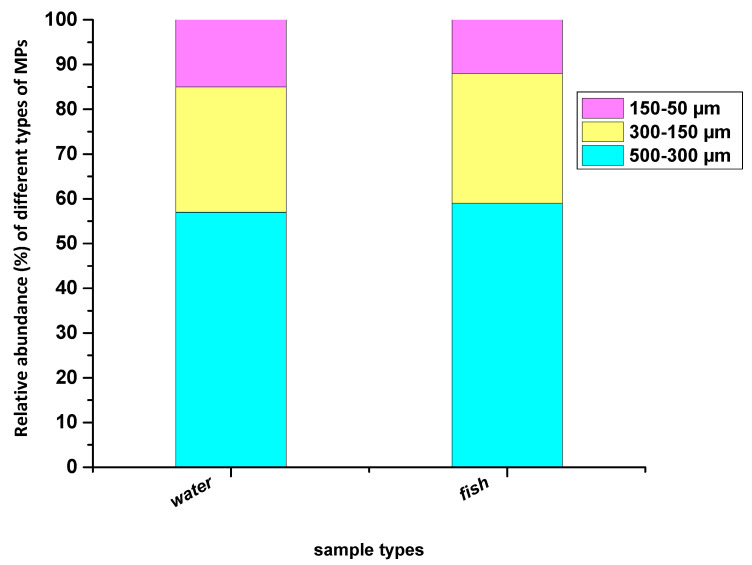
Relative abundance of identified MPs of three size classes that recovered from the surface water and fish GIT samples.

Based on the size, the major fraction of these detected MPs in the surface water were below 500 µm, and the total percentage of 300–500 µm particles was 57%. The mean concentrations of MP in the range of 150–300 µm and 50–150 µm size range were 28% and 15%, respectively (Figure 3). The same pattern of larger particles (300–500 µm) dominancy was recorded in the fish samples, wherein the mean percentage of MPs were 59%, 29%, and 12% for the 300–500 µm, 150–300 µm, 50–150 µm size class, respectively (Figure 3). The possible reasons for the higher MP size (500–300 µm) detections in our study could be that there are more large-sized MPs discharged into the surface water. The colonization of microorganisms (e.g., algae) and the adsorption of solid particles may increase aggregate sizes of MPs or small particle sizes of MPs tend to be underestimated due to limitations in separation, digestion, identification, and quantification methods [70,71]. Several published works have reported a remarkable amount of MP particles larger than 300 µm in fish GIT [52,53,70,72].

**Figure 4:**
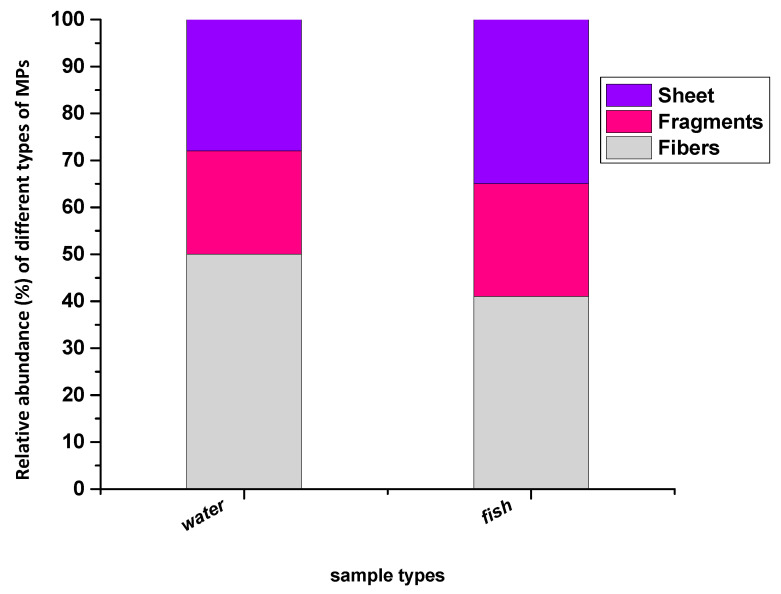
Relative abundance of three major particle shapes of the identified MPs recovered from the surface water and fish GIT samples.

**Figure 5:**
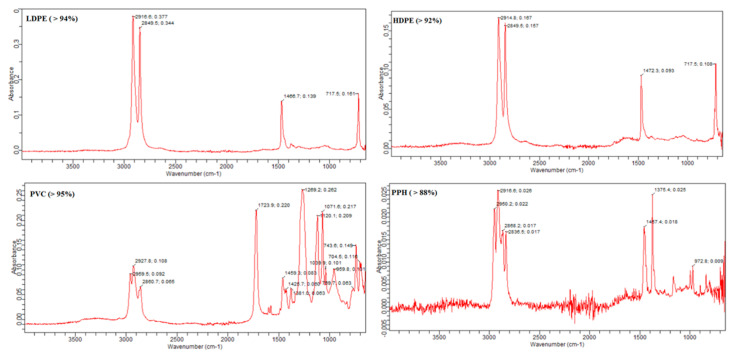
Detected polymer types in the current study.

Based on shapes, only three major shapes of MPs (fibers, sheets, and fragments) were detected from the water and fish samples. The fiber was the most abundant shape in surface water and fish samples, and fragments were the least abundant. The fraction of these fibers, sheets, and fragments of MPs in the surface water were recorded at 50, 28, and 22%, respectively (Figure 4). In fish GIT, fibers, sheets, and fragments were 41, 35, and 24%, respectively (Figure 4). Several reports on MPs found fibers as the most abundant types of MPs [57,67,69,70,71,72,73]. As the shapes of MPs depend on the originating sources of these particles, these fibers come from many sources, such as clothing, fishing, and industrial discharge into water bodies. In the present study, the shapes of MP particles observed were fibers, fragments, and sheets. No other shapes of MPs were seen, such as foam, filament, beads, etc. The main source of MP in the present study area was fishing activities, camping, and food wrappers disposed by tourists in the vicinity of the lake [57,67]. Beads and foams were absent in these samples. The primary MPs present in the studied area are facial scrubs and other skincare products. The chemical composition of the polymer type was confirmed by FTIR spectroscopy (IRTracer-100, Shimadzu, USA, Thermo Fisher Scientific Inc., USA software) [50]. The MP particles were placed on the ATR sensor, and their absorbance peaks were recorded. Furthermore, the comparison was made between recorded and standard peaks; thus, the composition of the particles was determined based on the peak similarity index. Comparatively larger particles with a range of 300–500 µm were tested as a representative of all particles. The low-density polyethylene (LDPE) (44.4%) and polyvinyl chloride (PVC) (30.5%) were the highest abundant types of polymer in the analyzed samples (Figure 5). Other types of polymers detected in the water and fish samples were polypropylene homopolymer (PPH) (19.4%) and high-density polyethylene (HDPE) (5.5%) (Figure 5). Other studies also have reported these polymers as a major type in their findings of MPs [50]. Mostly LDPE and HDPE polymers are used in packaging like foils, containers of milk, shampoos, oils, and soap bottles, household utensils such as trays, plates, cups, wires, PVC, electrical, electronic equipment, tour tents, and water pipes [52]. PPH is used to create packaging for a variety of products, including battery covers, pump components, structural tanks, etc. [74]. When all these plastic products are dumped into or close to water bodies, these utensils break down into small fragments. Disposable plates, cups, water bottles, food wrappers, and other items were frequently seen in the vicinity of the lake and the lake itself, left behind by the tourists. As a result, it was determined that the identified polymers in this study were related to their prospective use in the area under investigation.

## 4. Conclusions

The main findings of the present study showed that MPs have reached into higher altitudes in remote areas due to tourism activities. A considerable amount of MP particles was evident in the surface water of the Lake Mahodand and a trout fish in Kalam, Pakistan. Fibers were the most abundant types of MPs both in surface water and fish samples, and particles in the range of 500–300 µm were dominant in all samples. Low-density polyethylene (LDPE) was the most abundant type of polymer in the analyzed samples; other types of polymers detected in the water and fish samples were polyvinyl chloride (PVC), polypropylene homopolymer (PPH), and high-density polyethylene (HDPE). Our freshwater bodies are vulnerable to plastic load even in the absence of industrial impact at higher altitudes due to the rush of tourists and non-ecofriendly tourism activities, which can lead to several abnormalities in aquatic animals. Humans are also exposed to these MPs through their diet, which might cause many health issues. Thus, it is suggested to promote awareness about the harmful effects of plastics and guide the tourists to make their tours ecofriendly, to save natural resources and habitats from destruction.

## Figures and Tables

**Table 1:** Relative abundance of different sizes and shapes of MPs recovered.

	MPs Detected	Size of MPs			Shapes of MPs		
		500–300 µm	300–150 µm	150–50 µm	Fibers	Sheets	Fragments
**Surface water samples**							
1	2	1	0	1	1	1	0
2	3	2	1	0	3	0	0
3	0	0	0	0	0	0	0
4	3	1	2	0	1	2	0
5	2	0	2	0	1	0	1
6	1	0	0	1	1	0	0
7	0	0	0	0	0	0	0
8	3	3	0	0	1	1	1
9	4	4	0	0	1	2	1
10	3	2	1	0	1	2	0
11	3	2	0	1	3	0	0
12	4	3	0	1	1	1	2
13	0	0	0	0	0	0	0
14	5	0	5	0	1	3	1
15	3	0	0	3	3	0	0
16	1	0	1	0	1	0	0
17	2	2	0	0	0	1	1
18	0	0	0	0	0	0	0
19	3	3	0	0	0	0	3
20	4	3	1	0	4	0	0
**Fish GIT samples**							
1	3	1	2	0	0	2	1
2	2	1	1	0	1	1	0
3	0	0	0	0	0	0	0
4	1	1	0	0	1	0	0
5	3	3	0	0	1	1	1
6	1	0	1	0	1	0	0
7	1	0	0	1	1	0	0
8	3	2	0	1	2	1	0
9	1	0	1	0	0	0	1
10	2	2	0	0	0	1	1

## Data Availability

All data generated or analyzed during this study were included in this published article. All the raw and analyzed data will be available from the corresponding author based on reasonable demand.
